# The Effect of Mechanical Massage and Mental Training on Heart Rate Variability and Cortisol in Swedish Employees—A Randomized Explorative Pilot Study

**DOI:** 10.3389/fpubh.2020.00082

**Published:** 2020-03-19

**Authors:** Willeke Van Dijk, Anja C. Huizink, Jasmin Müller, Kerstin Uvnäs-Moberg, Anette Ekström-Bergström, Linda Handlin

**Affiliations:** ^1^Department of Clinical, Neuro and Developmental Psychology, Vrije Universiteit Amsterdam, Amsterdam, Netherlands; ^2^School of Health and Education, University of Skövde, Skövde, Sweden; ^3^Section of Anthrozoology and Applied Ethology, Swedish University of Agricultural Sciences, Uppsala, Sweden; ^4^Department of Health Sciences, University West, Trollhättan, Sweden

**Keywords:** stress, mental training, heart rate variability, cortisol, systolic blood pressure, mechanical massage

## Abstract

Work-related stress is relatively common in modern society and is a major cause of sick-leave. Thus, effective stress reducing interventions are needed. This study examined the effects of mental training and mechanical massage, on employee's heart rate variability (HRV) and plasma cortisol at their workplaces. Moreover, it was investigated whether baseline systolic blood pressure (SBP) can explain differences in effectiveness of the intervention. Ninety-three participants from four workplaces were randomly assigned to one of the five programs: (I) Mechanical massage and mental training combined, II) Mechanical massage, III) Mental training, IV) Pause, or V) Control. HRV and plasma cortisol were measured at baseline and after 4 and 8 weeks. SBP was measured at baseline. On the reduction of cortisol levels, a small effect of the mechanical massage program was found, whereas no effect was found for the other programs. None of the programs showed any effect on HRV. Nonetheless, when the level of systolic blood pressure was taken into account, some small beneficial effects on HRV and cortisol of mental training and the mechanical massage were found. This exploratory pilot-study provides useful information for future studies that aim to reduce stress among employees.

## Introduction

Work-related stress is one of the most prevalent forms of stress in modern society and it has personal and economic effects as it is a major cause of sick-leave. The high numbers of sick leave days due to stress highlight the need for effective interventions to reduce stress at workplaces (EU-OSHA; 2009). High psychological and emotional demands at work with low control abilities may lead to high levels of physiological and perceived stress which, if people do not take time to recover, can lead to an overactive stress-system (1). Biological indicators that can be used to assess individual's physiological stress level are cortisol and heart rate variability (HRV). Cortisol is a stress hormone that is secreted as a response to stress by the hypothalamic-pituitary-adrenal axis (HPA-axis), and can therefore be used as an index of stress. HRV, defined by the change in time interval between two consecutive heart beats, provides an indication of adaptability of the autonomic nervous system (2). These indicators can therefore serve as indicators when investigating the effectiveness of stress management interventions. Two stress management methods that have been applied to enable the body and mind to rest and recover are massage and mental training. Manual massage therapy has been shown to be effective in decreasing stress as indicated by a reduction of physiological outcomes, such as blood pressure (3, 4), and cortisol levels (4). This is partly supported in a meta-analysis, in which was reported that single applications of massage resulted in, among other outcomes, reduced blood pressure and heart rate, but not cortisol levels (5). Another method that has been used to reduce stress is mental training, which teaches people techniques to increase mental relaxation. Evidence on the effectiveness of mental training on physiological stress are scarce, however, one study showed a significant decrease in plasma cortisol in people who participated in a mental training intervention when compared to a control group (6). In a randomized controlled pilot study, the effects of massage therapy and mental training, separately or combined, have been examined in Swedish employees (7, 8). In this study a mechanical massage was used instead of the more commonly used manual massage. In contrast to the previously described studies that examined short term effects of massage and mental training programs, Muller et al. (7, 8) performed a randomized controlled study that examined the long term effects of mechanical massage and mental training, used both separately and in combination. In these two papers some positive effects of both the interventions have been described. In Muller et al. (7), positive effects on anxiety, stress susceptibility and detachment were found for both mental training and mechanical massage, separately and in combination. Regarding physiological effects, the mechanical massage significantly reduced heart rate and systolic and diastolic blood pressure, and increased fingertip temperature of the employees. Moreover, they showed that mental training significantly decreased employees' heart rate (8). The current paper further explores outcomes of this study and examines whether the mechanical massage and mental training, either separate or in combination, has an enduring effect on employee's heart rate variability (HRV) and plasma cortisol. It is expected that HRV increases and cortisol levels decrease as a result of the interventions that aim to reduce stress. Furthermore, it will be examined whether participant's baseline stress levels, as indicated by their initial systolic blood pressure (SBP), can have an influence on the effectiveness of the interventions.

## Method

### Participants

Participants were recruited from four workplaces located in the south-west of Sweden. We randomly allocated 93 participants who signed a written informed consent to one of the five study groups using sealed envelopes: (I) Massage and mental training combined (sitting in the armchair receiving mechanical massage while listening to a mental training program, *n* = 19), (II) Massage (sitting in the armchair receiving mechanical massage, *n* = 19), (III) Mental training (sitting in the armchair listening to the mental training program, *n* = 19), (IV) Pause (sitting in the armchair but not receiving mechanical massage nor listening to the mental training program, *n* = 19), or (V) Control (not sitting in the armchair at all, *n* = 17). Workplaces and participants were included if they had no prior experience with the armchair and/or mental training. Only healthy employees (self-reported) were included and employees who were pregnant, suffering from influenza, colds, fevers, or who had a skin or kidney disease, were excluded from participation. Participants should work between 75 and 100% within the concerning company. If participants were working <100%, the reason for this should not be stress-related issues.

### Procedures

The total study period was 8 weeks and participants in groups I–IV were instructed to attend three times a week to the assigned 15 min program, by preference between 1 and 4 pm. This study follows the Consort recommendations (7, 8) and is registered in Australian New Zealand Clinical Trials Registry http://www.anzctr.org.au/; ACTRN12615000020583, Date of registration: 15/01/2015.

#### Armchair

The armchair (Recovery Chair, Promas Method^TM^) is equipped with a mental training (verbal instructions via headphone) and a mechanical massage through which the neck, shoulders, back, and calves can be massaged. Groups I-IV were seated in the armchair while they received different programs. Participants in group V (control group) did not use the armchair.

The participants in the groups that received mechanical massage either listened to music (group II) or listened to the mental training (group I) while they were getting the massage. The mental training included verbal instructions, exercises, and soft background music and covered different topics each week in the following order: 1 “Recovery,” 2 “Mindfulness,” 3 “The way to a better and deeper sleep,” 4 “Reduce the negative stress,” 5 “Learn to think positively,” 6 “Increase your mental strength,” 7 “How to get a greater enjoyment of life” and eight “Recovery.”

The pause and control group neither got the massage nor the mental training. The pause group was asked to take a break from their regular work during which they had to sit in the armchair for 15 min doing nothing. The participants in the control group were instructed to continue with their work as usual with no break. More details on the procedures of the different conditions are described elsewhere (7).

### Measures

Measurements took place during three sessions; at baseline (before randomization), after 4 weeks, and after 8 weeks (end of study). All data collection was performed at the employees' workplace during working hours by a well-trained and experienced nurse and researcher. Data were collected on days that participants did not use the programs, in order to test long term effects of the programs. The total time of each session was 30 min during which blood collection, HRV, and blood pressure measurements took place. HRV was measured after a 30-min resting period. The participants were in seated position during all measurements. Because of the known circadian variation of cortisol levels, all data collection, including blood sampling, were performed before lunch between 9:00 and 12:00.

#### Dependent Outcomes

Heart rate variability (HRV) was determined using Biocom Technologies Heart Rhythm Scanner (Biocom 4000, Biocom Technologies, Poulbo, USA) software for 5 min. HRV data were obtained from electrocardiograph (ECG) with electrodes placed on the participant's wrists, and data were manually inspected for errors. The average HRV from the ECG was calculated for each of the occasions. The root mean square of successive differences (RMSSD) between consecutive heart beats, one of the time-domain variables which reflects vagal tone (2), were used as a measure of HRV (RMSSD; European-Society-of-Cardiology, 1996). Cortisol levels were obtained from blood samples collected at each measurement session. First, blood was collected in pre-chilled EDTA tubes and kept on ice until centrifugation. After that, plasma was taken and stored at −20°C until analysis. Plasma samples were processed using the Cortisol ELISA kit (Enzo Life Sciences Inc., Farmingdale, NY).

#### Independent Outcomes

Blood pressure was measured with an automatic manometer (Omron M6 Comfort, Omron Healthcare, Hoofddorp, the Netherlands). This device was connected to the left arm of the participants and placed in line with the heart.

### Ethical Considerations

The study was approved by the Regional Ethics Committee of the University of Gothenburg, Sweden (ref.nr: 980-12) and was carried out in accordance with the Declaration of Helsinki. The participants were allowed to end participation at any time and were informed that the data would only be available for the main researchers and would not be disclosed to employers. Participants assigned to the control group were able to use the armchair after the study period.

### Statistical Methods

We performed all statistical analyses with SPSS (version 24.0). To establish normal distributions of HRV and cortisol data, we log-transformed the data. We conducted One-way ANOVA tests and Chi-square tests to compare background characteristics of the groups. To compare baseline group values of HRV and cortisol, we used One-way ANOVA tests. We implemented linear mixed models (LMM) to analyze changes over time for HRV and cortisol separately. The dependent variables were HRV and cortisol measures (logRMSSD, logCortisol), with group (I–V) as independent variable. The measurements at the three time points (baseline, after 4 weeks, after 8 weeks) were the repeated measures. The fixed effects were group, time, and the interaction between group and time to test for differences between the groups over time. We conducted *t*-tests for each group to test for changes of HRV and cortisol between the different time points. Pearson's r was computed for HRV and cortisol for each group separately to analyze the relationship between an individual's baseline SBP and the change (Δ) between start and end of the interventions for the two outcome variables.

## Results

**Figure 1:**
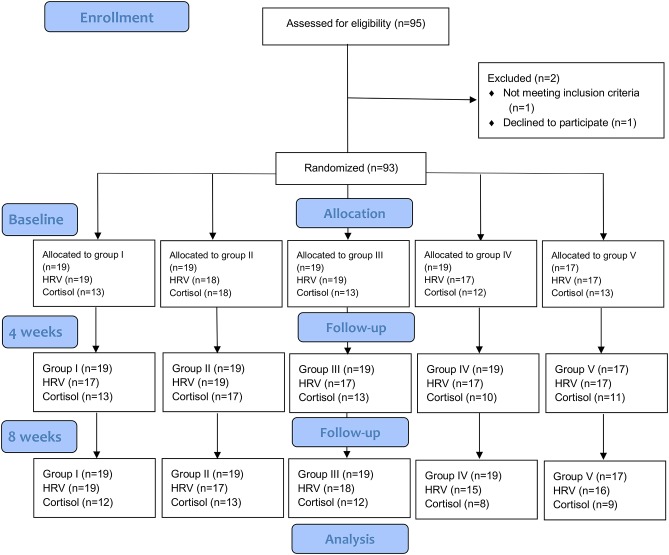
CONSORT flow diagram. Groups: I = massage and mental training, II = massage, III = mental training, IV = pause, V = control. CONSORT, Consolidated Standards of Reporting Trials; HRV, Heart Rate Variability.

The CONSORT flow-chart of participant recruitment can be found in Figure 1. Table 1 presents means and standard deviations of the continuous variables and frequencies of the categorical variables. The total sample of 93 participants consisted of 69 women (74.2%) and 24 men (25.8%) with a mean age of 47.6 years (SD = 9.84). The participants of the five study groups did not differ significantly in age (*F*_(4,88)_ = 0.54, *p* = 0.71).

**Table 1:** Background characteristics of the five study groups (*n* = 93) and baseline measures of systolic blood pressure. Values are presented as means (SD) or proportions.

**Characteristic**	**Groups**					**Comparisons**	
	**I (*n* = 19)**	**II (*n* = 19)**	**III (*n* = 19)**	**IV (*n* = 19)**	**V (*n* = 17)**	**χ^2^/F**	**ρ**
**Age**						0.54	0.71
Mean (SD)	50.4 (8.4)	47.5 (12.1)	46.5 (9.1)	47.9 (9.2)	46.7 (19.5)		
**Gender**						1.99	0.74
Women *n* (%)	16 (84.2)	15 (78.9)	13 (68.4)	13 (68.4)	12 (70.6)		
Men *n* (%)	3 (15.8)	4 (21.1)	6 (31.6)	6 (31.6)	5 (29.4)		
**Marital status**						5.01	0.75
Single *n* (%)	3 (16)	3 (16)	2 (10)	2 (10)	2 (11)		
Partner/married *n* (%)	15 (79)	16 (84)	17 (89)	17 (89)	14 (82)		
Living apart/other *n* (%)	0	0	0	0	1 (6)		
**Education**						4.67	0.96
Compulsory school *n* (%)	1 (5)	1 (5)	1 (5)	0	1 (6)		
Senior high school *n* (%)	5 (26)	3 (16)	2 (10)	4 (21)	2 (11)		
Higher education *n* (%)	2 (10)	3 (16)	2 (10)	3 (16)	1 (6)		
University *n* (%)	11 (58)	12 (63)	14 (74)	12 (63)	13 (76)		
**SBP**						0.19	0.94
Mean (SD)	128.62 (17.36)	125.89 (16.69)	127.74 (20.50)	127.00 (23.56)	123.59 (14.09)		

*Groups: I = massage and mental training, II = massage, III = mental training, IV = pause, V = control; SD, standard deviation; SBP, Systolic blood pressure measured at baseline*.

### Effect of the Programs on HRV and Cortisol

We had complete data on HRV from 79 participants. The five groups did not differ significantly in baseline levels of HRV (*F*_(4,85)_ = 1.39, *p* = 0.24). In our main analysis we found no differences in HRV over time between the five study groups. The results of the LMM analysis suggest that neither time, group, nor the interaction were significant predictors in explaining variance in HRV. We found no significant differences in HRV from baseline to 4 weeks, 4–8 weeks, and baseline to 8 weeks for none of the groups.

For cortisol, we had complete data of 43 participants. We found no significant differences on baseline cortisol levels (*F*_(4,64)_ = 0.23, *p* = 0.92). The results of the LMM analysis suggest that neither time, group, nor the interaction were significant predictors in explaining variance in cortisol. We found a significant decrease in cortisol in the massage group only between the fourth (*M* = 19.77, SD = 12.59) and the 8-weeks (*M* = 15.96, SD = 7.39) measurement (*t*(12) = 2.19, *p* < 0.05). The other measurements of the massage group were not significantly different, nor were the measurements of the other groups.

### Correlation Between SBP With HRV and Cortisol

To test if baseline SBP was correlated with the change (from start to the end of the intervention) in HRV and cortisol (Table 2), we computed Pearson's r for each group. We found no significant differences of baseline SBP between the groups (*F*_(4,85)_ = 0.19, *p* = 0.94) (Table 1). The results for change in HRV show a significant negative correlation with SBP in group II (*r* = −0.61, *n* = 15, *p* < 0.05) (Table 3), suggesting that only for the group that was offered the mechanical massage as only intervention, lower SBP was correlated with a stronger increase in HRV from baseline to the end of the intervention. In contrast, we found a positive correlation with cortisol and SBP in group III, suggesting that for the group that was offered the mental training as only intervention, lower SBP was correlated with less decrease in cortisol from baseline to the end of the study period (Table 3).

**Table 2:** Means and standard deviations of the ΔlogHRV and ΔlogCortisol for each group separately.

**Group**	**ΔHRV**	**ΔCortisol**
	**Mean (SD)**	**Mean (SD)**
I (*n* = 19)	−0.24 (0.63)	−6.09 (17.09)
II (*n* = 19)	0.17 (0.44)	3.09 (11.48)
III (*n* = 19)	−0.02 (0.30)	1.02 (9.13)
IV (*n* = 19)	−0.01 (0.54)	2.99 (11.44)
V (*n* = 17)	−0.03 (0.42)	−1.99 (9.28)

*Groups: I = massage and mental training, II = massage, III = mental training, IV = pause, V = control; SD, Standard Deviation*.

**Table 3:** Correlations of change (Δ) in HRV and cortisol, between start (baseline) and end of the intervention (after 8 weeks) with baseline SBP (group I to V) for each group.

**Parameter**	**Group**	**ΔHRV**	**ΔCortisol**
SBP	I	0.01	−0.01
	II	−0.61*	0.07
	III	0.08	0.75*
	IV	−0.004	−0.15
	V	−0.27	0.25

Groups: I = massage and mental training, II = massage, III = mental training, IV = pause, V = control; ΔHRV = HRV_8−weeks_–HRV_baseline_; ΔCortisol = Cortisol_baseline_–Cortisol_8−weeks_; *p < 0.05.

## Discussion

This small-scaled, randomized controlled pilot study explored whether an 8-weeks lasting mechanical massage and mental training intervention, separate or in combination, could have an effect on employees' HRV and cortisol levels. The main result of the study shows that there is no difference in change over time between the groups for HRV and cortisol. When baseline stress levels, as indicated by baseline SBP, were taken into account, participants with lower SBP who were offered mechanical massage showed more beneficial effects on HRV and of those with lower SBP who were offered mental training showed more beneficial effects on cortisol when compared with the participants in these groups who had higher SBP.

### Effects of the Stress Intervention Programs on HRV and Cortisol

When looking at the effects of the programs within the groups, no change in HRV between the three time points was observed in any of the groups. For cortisol, a significant decrease between the fourth and the 8-weeks measurement was found, only in the group that was offered the mechanical massage. When the five study groups were compared with each other, there were no differences in HRV and cortisol between the programs, neither at the three different time points, nor in the effects on HRV and cortisol over time. These results are not in line with our expectation that the mechanical massage, mental training, and the combined program, would be more effective in increasing HRV and decreasing cortisol when compared to both the control and pause group.

Some explanations can be provided for these unexpected results. Diego and Field (9), who also examined the effects of massage in healthy participants, distinguished between light and moderate pressure massage. Their findings showed that after light pressure massage an increase in sympathetic activity was found, whereas after moderate massage a shift from a sympathetic to parasympathetic response was shown, indicating higher levels of relaxation in the latter group. The participants in the current study could adjust the settings of the massage when desired. We assume that people would be able to relax more when they can choose a massage strength where they feel most comfortable with. However, variation in settings amongst the sessions within one participant could have caused different effects of the massage, like Diego and Field (9) found between light and moderate pressure massage. For example, if one would have chosen light massage at 1 day and hard massage at another day, the possible increase in sympathetic activity (due to light massage) and increase in parasympathetic activity (due to hard massage) could have canceled each other out. If this had happened in our study, then the interpretation of effect of the mechanical massage could have been underestimated. We did not assess which strength of the massage chair was used by our participants nor did we measure whether this strength was the same at each occasion they used the chair, which could be interesting for further investigation.

A study that found a positive effect of massage in healthy students, only showed improvement in HRV for individuals with higher stress sensitivity and not for individuals who were less responsive, as determined by the cold pressor stress test Díaz-Rodríguez et al. (10). Thus, when stress responsiveness of the participants was taken into account, massage was differentially effective among individuals. Individual differences in stress responsivity among participants might explain differences in effect of massage and the other programs of the present study as well, as this was not taken into account in this study. A possible reason why we did not detect differences in cortisol during the intervention period could be that the length of the intervention was just too short to achieve an effect. We found a significant decrease between the fourth and the 8-weeks measurement, but only in the group that was offered the mechanical massage. Johansson and Uneståhl (6) showed a positive effect of a 6-months mental training program, as indicated by a decrease in plasma cortisol after the intervention period. Possibly, a longer intervention period than the 2 months of this study is needed to achieve the desired effects of mechanical massage, mental training, and the combined program. It should be mentioned that the current study explored the lasting effects of mechanical massage and mental training program, as assessments were done at days that participants did not perform the program(s). This study did not focus on the acute or short term effects of the interventions, as the previous studies did (6, 9, 10). Positive long lasting effects of both mechanical massage and mental training on psychological as well as physiological outcomes have previously been demonstrated (7, 8). Muller et al. (7) showed positive effects of both mental training and mechanical massage, separately and combined, on anxiety, stress susceptibility and detachment. Muller et al. (8) showed beneficial effects on heart rate, blood pressure, and fingertip temperature after mechanical massage, and on heart rate only after mental training. When combined with the results of the current study, one could presume that the direct effects of mental training and mechanical massage on cortisol and HRV, if they would have been measured directly after the programs, were less stable over time when compared with other physiological measures, such as heart rate, blood pressure, and fingertip temperature. All in all, whether the effects of massage, either mechanical or manual, and mental training are transient or enduring, needs to be further investigated. To explore a possible temporal change in the effects of such programs, a design that enables testing both immediate and lasting effects is required.

### SBP and Effect of Programs on HRV and Cortisol

Furthermore, this study investigated whether individual's baseline SBP, an indicator of people's stress level, can explain differences in effectiveness to the programs between participants as measured by HRV and cortisol. In fact, blood pressure of individuals that is already at a low or healthy level does not need to be further reduced, which is in contrast to people with high blood pressure, who have a higher necessity to lower their blood pressure as hypertension is a risk factor for cardiovascular diseases (11). Therefore, stress interventions might be more relevant for people with high blood pressure. It was hypothesized that for these individuals, a higher positive change in HRV and a higher negative change in cortisol from baseline to the end of the study period would be found as a result of the programs, when compared to participants with lower blood pressure. The results are not fully in line with these expectations since it was only for the mechanical massage group that lower SBP was correlated with a stronger increase in HRV. For cortisol, the results suggest that only for the mental training group, lower SBP was correlated with a stronger decrease in cortisol from baseline to the end of the intervention. A possible explanation for these results could be that individuals with higher SBP are too stressed to relax through the mechanical massage and therefore a stress intervention like mechanical massage is not effective in lowering the stress levels in this specific group. If higher SBP is a true indicator of excessive perceived stress, mechanical massage could be recommended to be used for preventive purposes rather than to help people recover from already too much elevated stress levels.

### Strengths and Limitations

One of the strengths of this study is that it is one of the first to examine the effect of mechanical massage, without the need of a masseur, on physiological measurements. The use of a mechanical massage instead of a manual massage excludes the possible bias by therapist-effects and thereby enables to test the true effects of massage. Also, by including a pause group and a control group we were able to examine whether the stress reduction was due to the program(s), or whether a period of rest in between work is sufficient to achieve stress reductions. Moreover, as randomization took place within the participating companies, we reduced the possibility of confounding by differences among workplaces and therefore we provide a less biased evaluation of the stress intervention programs. The small sample and the associated restricted statistical power are limitations of this study as this may have led to an under- or overestimation of the effect of the interventions. Power calculations prior to the study suggested a sample size of 100 participants in each group [for a 30% reduction of cortisol levels in de experimental groups compared to pause and control (*b* = 0.8 and *a* = 0.05)]. Due to financial and time constraints, we were unable to achieve this required sample size. Due to the small sample size, we were also restricted in using covariates in our main analyses. A second potential short-coming is the lack of control for possible confounding variables such as variation in personality, coping style, as well as lifestyle or early life experiences among participants that may have influenced participants responses to the programs offered. These limitations should be taken into consideration when interpreting the results.

## Implications and Conclusion

Work-related stress is a major source of stress related problems in modern society and therefore interventions focusing on the modulation of stress at the workplace are needed. Our current findings suggest that a mechanical massage or mental training seems to be effective for individuals with lower SBP, perhaps implying that people with relatively low levels of stress may benefit from our programs in a preventative manner. In contrast, people with higher SBP at baseline, may need a longer or more intensive program to reduce their levels of stress. Thus, these stress intervention programs can be valuable for clinical practice as, when offered by employers at workplaces, it can help to keep employees healthy and prevent development of stress-related illnesses. Moreover, the programs examined in the current study might be less expensive and easier applicable when compared to other stress interventions such as manual massage. The current study also suggests that there is a small effect of the massage program on the reduction of cortisol. However, as this is a small-scaled exploratory pilot study, further research consisting of a larger sample, including covariates (normative stress, stress susceptibility, coping styles, early life experiences) and follow-up measurements are needed to confirm the possible effects and evaluate the long-term effects of stress interventions. Nonetheless, this exploratory pilot-study provides useful information for future studies that aim to reduce stress among employees.

## Data Availability Statement

The datasets analyzed in this article are not publicly available due to risk of compromising individual privacy. The application and the written consent forms approved by the Regional Ethical Review Board in Gothenburg, Sweden, states that the data will only be available to the researchers within the project. Requests to access the datasets should be directed to linda.handlin@his.se.

## Ethics Statement

The studies involving human participants were reviewed and approved by the Regional Ethics Committee of the University of Gothenburg, Sweden. The patients/participants provided their written informed consent to participate in this study.

## Author Contributions

JM, LH, AE-B, and WV were active in the design, data collection, and analysis. WV, LH, and AH were involved in the writing-up of the manuscript. All authors also read and approved the final manuscript.

### Conflict of Interest

The authors declare that the research was conducted in the absence of any commercial or financial relationships that could be construed as a potential conflict of interest.
